# New onset migraines after maxillary antrostomy for silent sinus syndrome treated with rimegepant: A rare case report

**DOI:** 10.1016/j.bjorl.2025.101580

**Published:** 2025-04-03

**Authors:** Aishwarya Aggarwal, Yasemin Akinci, Brian L. Gerhardstein

**Affiliations:** a Department of Neurology, Hackensack Meridian Health JFK University Medical Center, Edison, NJ, United States

## Introduction

Silent Sinus Syndrome (SSS) is a rare clinical entity characterized by progressive enophthalmos (posterior displacement of the globe in the orbit) and hypoglobus (globe displaced downward in the orbit) due to gradual collapse of the orbital floor with opacification of the maxillary sinus, in the presence of subclinical maxillary sinusitis. The cause of silent sinus syndrome is hypoventilation of the maxillary sinus due to obstruction of the ostiomeatal complex. This hypoventilation over a course of weeks to months results in resorption of gases into the capillaries of the closed sinus cavity, which creates a negative pressure. This leads to accumulation of secretions with chronic subclinical inflammation, resulting in maxillary sinus atelectasis. Inward bowing of maxillary sinus walls secondary to negative pressure buildup causes subsequent thinning of maxillary walls.^1^, ^2^ Individuals with syndrome may present to an ophthalmologist with diplopia, eyelid abnormalities (retraction, ptosis, absent crease), ocular asymmetry or dry eye.^3^

Both Computed Tomography (CT) and Magnetic Resonance Imaging (MRI) can be diagnostic for SSS. Imaging often demonstrates an opacified and hypoplastic maxillary sinus; a lateralized uncinate process; depression of the orbital floor and a blocked osteomeatal complex. The two main goals of treatment are to improve the maxillary sinus drainage and to restore normal orbital anatomy. A wide maxillary or middle-meatal antrostomy is performed to enlarge the maxillary sinus ostium. There are three approaches depending on the additional requirement for orbital reconstruction and its timing. The first approach is a combined single-stage operation where antrostomy and reconstruction are performed at the same time. The second type is a two-stage approach where orbital reconstruction is done 2–6 months after antrostomy. In the third approach no reconstruction is required as some patients improve with sinus surgery alone.^3^

## Case presentation

A 69-year-old female with no prior history of headaches was seen by otolaryngology for the evaluation of chronic rhinitis and ear problems that she had for a few years. The patient did not report any diplopia or other visual problems but did notice a cosmetic difference in her eyes. She underwent a diagnostic rigid nasal endoscopy which showed that her nasal septum was deviated to the right and her inferior turbinates were enlarged. She underwent a maxillofacial CT scan which showed right sided silent sinus syndrome with right hypoglobus. Patient underwent right maxillary antrostomy with removal of inflamed tissue.

Three weeks after the surgery she developed a headache which got progressively worse. She described the headache to be bifrontal and generally eight out of ten in intensity. Her headache lasted all day long and was severely debilitating. She also reported severe sensitivity to light, smell and sound. She was unable to look at her phone due to extreme light sensitivity. She became intolerant to high-pitched sounds and had constant sensation of pressure and pain in her ears. She denied any nausea or vomiting. She reported some improvement in headaches with rest and reported feeling best upon waking. She denied any changes in her vision. There was no relief in her symptoms with over-the-counter medications. She underwent a repeat CT scan of her maxillofacial region which demonstrated interval right nasal antrostomy with clearing of right maxillary sinus opacification. Her headaches were thus not thought to be secondary to the procedure and she was referred to neurology by her otolaryngologist.

Four months later she saw a neurologist and underwent a brain MRI, Magnetic Resonance Angiography (MRA) and Magnetic Resonance Venography (MRV) which were all unremarkable. The patient was prescribed Topiramate which was tapered from 25 mg to 75 mg. Patient reported no relief of her headaches with the medication and reported experiencing increased anxiety, difficulty processing information and hair loss while on the medication. She then stopped taking the medication after a few weeks.

She presented to our Neurology clinic about six months after the onset of her headaches and reported that she was severely debilitated. We decided to give her a trial of treatment with Rimegepant 75 mg every other day and advised her to follow up in a month.

Upon her follow up visit a month later, patient reported that there was a very significant improvement in her headaches and that she no longer had photophobia, phonophobia or osmophobia. She was able to resume her prior lifestyle after the medication.

## Discussion

In conclusion, this is a rare case of new onset migraine-like headaches after sinus surgery. It is known that extracranial structures of head such as skin, muscles, arteries, and periosteum can generate pain. Peripheral sensory afferents from structures of the head and neck (via the trigeminal nerve) converge with nociceptive fibres from intracranial vascular structures and the meninges. These afferents project from the trigeminal ganglion to the trigeminocervical complex within the brainstem. Ascending projections between the trigeminocervical complex and various cortical processing areas pass through the thalamus. Cortical projections from the trigeminocervical complex via the hypothalamus, midbrain, pontine and medullary nuclei are also present. Fibres innervating the dura release various neuropeptides on activation including neurokinin A, calcitonin Gene-Related Peptide (CGRP), Pituitary Adenylate Cyclase-Activating Polypeptide (PACAP) and these cause vasodilatation of dural and pial blood vessels which is thought to cause and maintain headache. We hypothesise that the periosteal irritation from the sinus surgery procedure led to the onset of migraine headaches through the pathway described above.^4^ Rimegepant is a CGRP receptor antagonist approved for the acute treatment of migraine with or without aura in adults, and for the preventive treatment of episodic migraines in adults. The response to Rimegepant as preventative medication in this patient supports the diagnosis of headaches as probable new onset migraines.^5^

## Conclusion

We report a rare case of SSS. Additionally, this case demonstrates how sinus surgeries can lead to migraine like headaches and efficacy of CGRP antagonists in the treatment.

## CRediT authorship contribution statement

Aishwarya Aggarwal: Writing the manuscript (First author).

Yasemin Akinci: Review of Literature (Second author).

Brian L. Gerhardstein: Supervision (Third author).

## Ethical considerations

An informed consent was obtained from the patient. Patient confidentiality will be maintained. No harm was done to any individual. It is an observation based on clinical practise.

## Funding source

None.

## Data sharing

Data sharing upon reasonable request to the authors. Patient’s personal information will not be shared.

## Declaration of competing interest

The authors declare no conflicts of interest.

## References

[1] Numa W.A., Desai U., Gold D.R., Heher K.L., Annino D.J. (2005). Silent sinus syndrome: a case presentation and comprehensive review of all 84 reported cases. Ann Otol Rhinol Laryngol.

[2] Pula J.H., Mehta M. (2014). Silent sinus syndrome. Curr Opin Ophthalmol.

[3] Thomas R.D., Graham S.M., Carter K.D., Nerad J.A. (2003). Management of the orbital floor in silent sinus syndrome. Am J Rhinol.

[4] Karsan N. (2024).

[5] Blair H.A. (2023). Rimegepant: a review in the acute treatment and preventive treatment of migraine. CNS Drugs.

